# Seeing nonspectral colors with single wavelength stimulation in two-photon vision

**DOI:** 10.1364/BOE.575139

**Published:** 2025-10-30

**Authors:** Linus Emmerich, Pedro Gil, Silvestre Manzanera, Juan Tabernero, Pablo Artal, Christina Schwarz

**Affiliations:** 1 Institute for Ophthalmic Research, University of Tübingen, Tübingen, Germany; 2 Graduate Training Center of Neuroscience, University of Tübingen, Tübingen, Germany; 3 Electromagnetismo y Electrónica, Universidad de Murcia, Murcia, Spain; 4 Laboratorio de Óptica, Universidad de Murcia, Murcia, Spain; 5 Center for Optical Technologies, Aalen University, Aalen, Germany

## Abstract

Pulsed near-infrared (NIR) lasers can be perceived as light of approximately half their wavelength due to the process of two-photon (2P) absorption. For high intensities of light, single-photon (1P) absorption can still be perceived beyond 700 nm, so that the overall color perception in this region is the result of a mix of 1P and 2P absorption. In this study, color matching experiments were performed with seven laser wavelengths between 730 and 920 nm to investigate the interaction between 1P and 2P absorption and the range of colors that can be created by changing the laser power and repetition frequency of a ns-pulsed laser. We recorded color matches in a range from pure red for shorter wavelengths across shades of purple up to pure blue colors for the longest wavelengths, showing that nonspectral (purple) colors can be created using only one stimulating wavelength in the NIR. Changes in hue could be observed between wavelengths of 850 nm and 920 nm when laser power or repetition frequency were modified, with the highest color shifts occurring between 880 nm and 900 nm.

## 1. Introduction

It has been known for a long time that humans can perceive infrared (IR) light created with pulsed lasers way beyond the spectral range of 400 - 780 nm, which is typically referred to as the visible spectrum. Different studies have shown that wavelengths of up to 1200 nm can be seen through the process of 2P absorption [1–6]. Properties like color, brightness and perception threshold in the NIR [7–11], as well as 2P absorption in conjunction with ocular diseases [12–14] and the application of 2P microperimetry [15–17] have been examined. Triggering photoreceptor chromophore isomerization by two-photon absorption causes those stimuli to appear roughly at half the wavelength of the infrared laser radiation [18,19]. While this correlation is accurate for wavelengths beyond 900 nm well above the perception threshold, shorter wavelengths create a hybrid color perception by combining conventional single-photon excitation of photoreceptors as well as 2P vision [20]. This results in a mix of single-photon red perception and a blue perception caused by 2P stimulation, creating different shades of purples. This set of colors cannot be recreated conventionally with a single monochromatic light source, but requires at least two sources at both ends of the visible spectrum. The interaction introduces a unique opportunity to modulate colors in the NIR using a single stimulating wavelength. Capitalizing on the nonlinear properties of 2P absorption, it is possible to manipulate the ratio of 1P and 2P excitation by adjusting laser parameters such as wavelength, laser power, and repetition frequency. This contradicts Grassmann’s laws, a set of principles describing the linear and additive properties of color mixing and metamerism published in 1853 [21]. Alongside the principles of symmetry, transitivity and additivity, proportionality is given as a key property of color matching, meaning that two metamers persist when amplified/attenuated by a constant factor [22]. As a change in luminosity resulting from a change in laser power or repetition frequency also entails a change in hue in the regime of mixed 1P/2P stimulation, metamerism with a conventional 1P stimulus does not sustain.

This study aims to investigate the spectral range of hybrid 1P/2P vision and the changes in perceived hue that can be generated by modulating laser power and repetition frequency.

## 2. Methods

### 2.1. Experimental setup

**Fig. 1.**
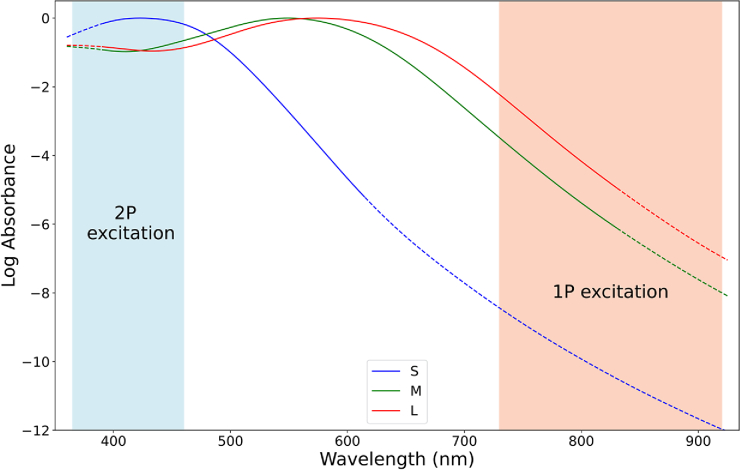
Absorption spectra of cones, wavelength ranges of conventional stimulation and 2P excitation are marked in red and blue. Spectra were generated using the formulae provided by Stockman and Rider [25].

A retinal projection display [23] was used to perform psychophysical experiments. A supercontinuum pulsed laser (SuperK COMPACT, NKT Photonics, Birkerød, Denmark) provided IR stimuli of 730, 760, 800, 820, 850, 880, 900 and 920 nm (FWHM: 10-40 nm) with a pulse length of 1 ns and repetition frequencies of 3 and 15 kHz. Average laser powers of 10, 20 and 30 μW were used for both repetition frequencies, although 30 μW of laser power were not feasible in many cases and had to be omitted in the analysis. An overview of all analyzed laser parameters can be found in Table 1, Fig. 1 shows the ranges of 1P and 2P stimulation covered in the experiments. Using a pair of galvo scanners, a square of 20x20 arcmin was projected onto the retina. For color matching, an OLED screen (SXGA, eMagin Corporation, Hopewell Junction, NY, USA) was projected right next to the laser stimulus, adjusted to the same shape and size as that of the IR stimulus. No additional accommodation target was used. Participants were positioned with custom-made bite bars on an XYZ translation stage. Detailed information on the experimental setup can be found in [20,23].

Equation 1 shows the influence of different system parameters on 2P absorption that was first described by Denk, Strickler and Webb with regard to 2P laser scanning fluorescence microscopy [24] and can be transferred to visual stimulation. It describes the number of photons absorbed per molecule per pulse $\left(N_{a}\right)$ , with the two-photon cross-section $\sigma_{2ph}$ , the average laser power $p_{avg}$ , pulse duration $\tau_{p}$ , repetition frequency $f_{p}$ , numerical aperture NA , reduced Planck’s constant ℏ , the speed of light *c* and the laser wavelength *λ*:

(1) $$N_{a}=\sigma_{2ph}\frac{p_{avg}^{2}}{\tau_{p}f_{p}^{2}}\;{\left(\frac{NA^{2}}{2\hbar c\lambda }\right)}^{2}$$

**Table 1.** All feasible combinations of parameters were tested in the measurements

	Laser parameters

Repetition rate, kHz	3, 15
Laser power, μW	10, 20, (30)
Wavelengths, nm	730, 760, 800, 850, 880, 900, 920

### 2.2. Color matching procedure

Five healthy volunteers between the age of 26 and 51 without known ocular diseases were included in the study. Non-emmetropic participants performed the psychophysical experiments with their usual correction (spectacles/ contact lenses). Color matching was performed in a dark room with all remaining light sources from electronic components covered and after a dark adaptation of 20 minutes prior to the experiments. Participants were fixed in the setup with custom made bite bars and adjusted the OLED screen themselves so that it would appear right next to the stimulus of the pulsed laser. With both stimuli next to each other, participants used a keyboard to adjust the OLED by changing hue, saturation and brightness. For each combination of wavelength, laser power and repetition frequency, participants performed three trials of colormatching without a time limit, HSV values were saved for each colormatch before resetting the OLED settings for the next trial. The experiment was performed in two sessions on separate days, covering four wavelengths on the first date and the remaining three wavelengths on the second date. Laser safety calculations were performed according to the ANSI Z136.1-2022 standard [26] and the publication by Delori et al. focusing on ocular safety in ophthalmic devices [27]. All tests were performed after obtaining informed consent from participants. The measurements complied with the Declaration of Helsinki and were approved by the Ethics Committee of the University of Murcia (Spain).

### 2.3. Spectral measurements

After the color matching experiments, all saved OLED settings were measured with a spectrometer (USB4000, Ocean Optics, Largo, FL, USA) directly in front of the screen. Additionally, pure red, green, and blue screens were measured to determine the gamut of the OLED. To consider potential color changes caused by the optical elements, measured spectra were propagated through the lens, mirror and beamsplitter between the screen and the participant using the reflection/transmission data provided by the manufacturer.

### 2.4. Data analysis

**Fig. 2.**
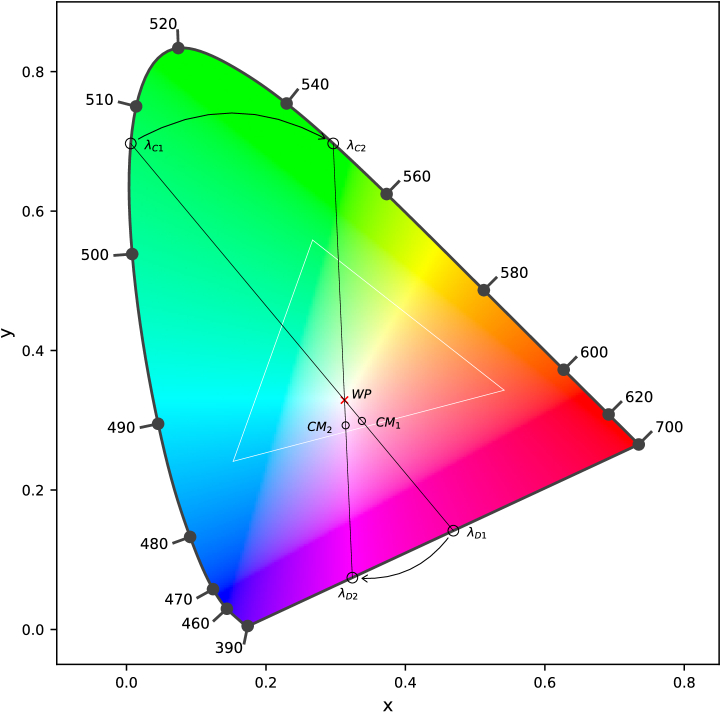
Two colormatches (CM_1_, CM_2_) in the CIE 1931 colorspace, plotted with dominant wavelength $\left(\lambda_{D1},\lambda_{D2}\right)$ on the line of nonspectral purples and the complementary wavelength $\left(\lambda_{C1},\lambda_{C2}\right)$ on the spectral locus, connected through the whitepoint (WP). The white triangle shows the gamut of the OLED.

Propagated spectra were converted to CIE 1931 chromatic coordinates before averaging the three trials for each set of parameters and participant. As the color coordinates are spread out along the nonspectral purple line between red and blue, the dominant wavelength cannot be calculated in the majority of cases. In order to define the colorshift between different parameters, the complementary wavelengths were used instead as illustrated in Fig. 2. They are determined by connecting the color coordinates CM_1_ and CM_2_ with the white point (WP; x =0.33, y=0.33) and extending these lines to intersect the spectral locus. Instead of using the intersecting points closest to the measurement coordinates that represent the dominant wavelengths $\lambda_{D1}$ and $\lambda_{D2}$ , the intersecting points closest to the white point $\lambda_{C1}$ and $\lambda_{C2}$ are used for further analysis. As a measure of change in perceived color, the shift in complementary wavelength was calculated for each participant and parameter setting. All conversions and calculations were performed in Python.

## 3. Results

**Fig. 3.**
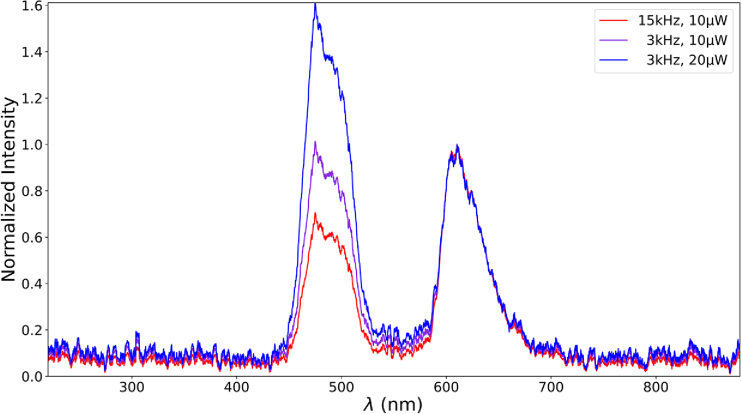
Three averaged spectra measured from the OLED, showing color matches for different combinations of laser power and repetition frequency (i.e. two-photon efficiency) across all subjects at a stimulation wavelength of 880 nm. Spectra are normalized for the red peak at ∼ 620 nm.

**Fig. 4.**
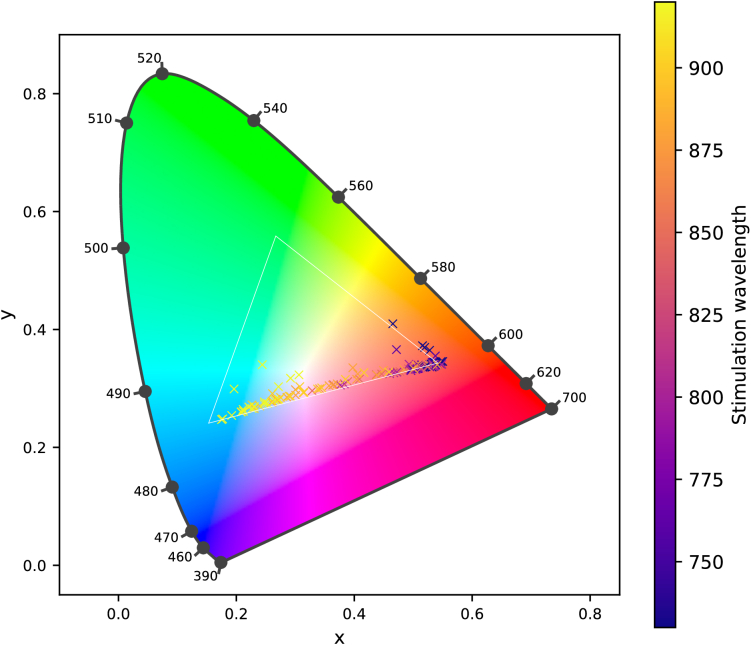
Colormatches scatter along the purple line of the CIE 1931 chromaticity diagram, inside the gamut of the OLED.

Measured spectra from the OLED screen show different ratios of blue and red peaks with no significant green component. Examples can be seen in Fig. 3. After converting spectra to CIE 1931 chromatic coordinates, all measured color percepts are located at the purple line of the OLED gamut, as shown in Fig. 4. Separate figures for each wavelength can be found in Supplement 1, providing information about the laser parameters of each data point. Shorter wavelengths with a higher sensitivity for 1P absorption can be found in the red corner of the gamut, while longer wavelengths with low sensitivity for 1P absorption are located in the blue corner of the gamut. Figure 3 displays this formation very clearly, showing two main peaks in the red and blue wavelengths of the spectra that vary in relative intensity. Between 850 nm and 920 nm, a shift in complementary wavelength can be observed with highest mean shifts ranging from 15 nm up to 51 nm for different changes of laser power and repetition frequency. There is a high variability between subjects both in the location of colormatches and in the shifts in complementary wavelength between trials with changed parameters.

**Fig. 5.**
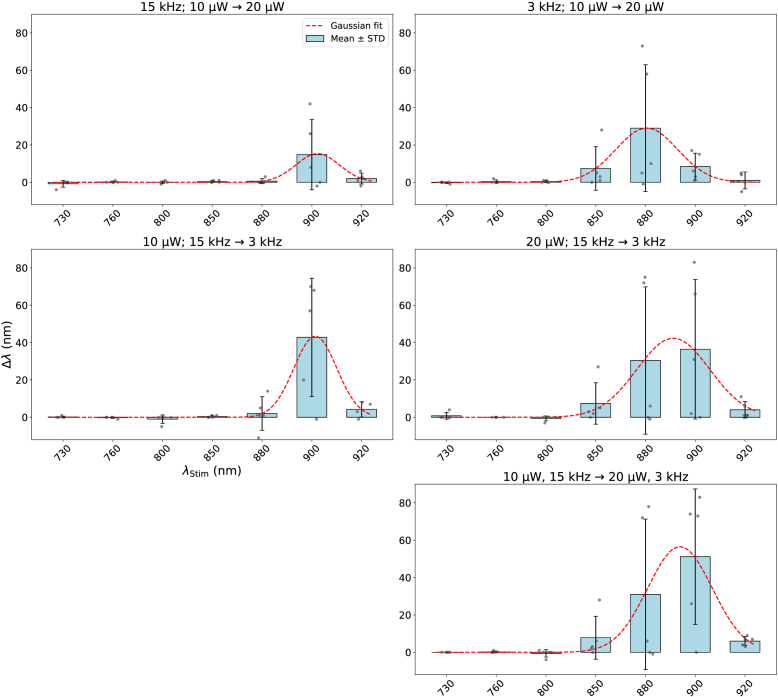
Mean shifts in complementary wavelength. Top row: Wavelength shift with changes in laser power (10 μW to 20 μW). Middle row: Wavelength shifts with changes in repetition frequency (15 kHz to 3 kHz). Bottom row: Wavelength shift with changes in laser power and repetition frequency. The red line represents a gaussian fit to the data.

The shifts for all parameter changes and laser wavelengths can be observed in Fig. 5. The top row shows the shifts in complementary wavelengths when increasing laser power from 10 μW to 20 μW for both repetition frequencies, while the middle row shows the shift when decreasing repetition frequency from 15 kHz to 3 kHz. The combined effect of increased laser power and reduced repetition frequency (bottom row) appears more pronounced than isolated effects, but the effects do not add up. For parameters resulting in higher 2P efficiency (right side), the peaks of the blue shifts are located at shorter wavelengths. Unfortunately, a laser power of 30 μW was not feasible in many cases. For short wavelengths, the laser was uncomfortably bright for participants, though within laser safety limits. For long wavelengths, laser power was not high enough to provide 30 μW of average power within the narrow bandwidth of 10 nm. Due to the low number of measurements recorded with a laser power of 30 μW, they were excluded from the analysis.

**Fig. 6.**
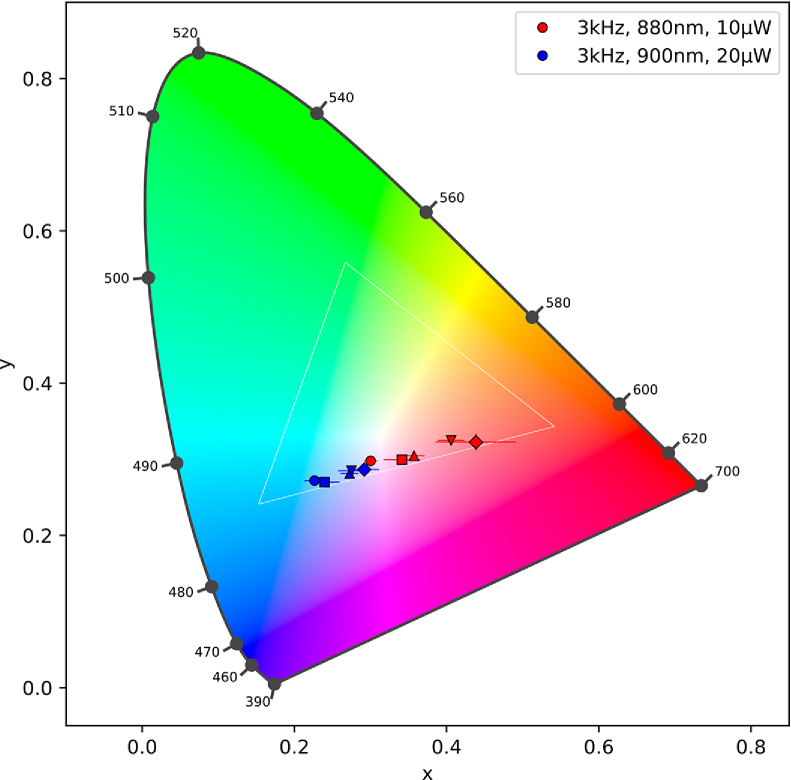
Colormatches of individual participants show large variability, whereas a blueshift is generally visible. Each marker represents one participant.

## 4. Discussion

In the psychophysical experiments, colormatches were recorded from pure red to purples to pure blue for different combinations of laser wavelength, repetition frequency and laser power. As the red component of the IR color percept is triggered by conventional 1P absorption, measurements located in the red corner of the gamut evoke dominantly 1P excitation and a low quota of 2P excitation in direct comparison. In contrast, measurements located in the blue corner of the gamut can be characterized by inducing a high amount of 2P excitation and a negligible amount of 1P excitation, while measurements in the purples represent a mixed ratio of 1P and 2P stimulation. For the laser parameters used in these experiments, we can conclude that there is negligible 2P absorption for wavelengths below 850 nm, while there is no substantial contribution of 1P absorption to the color perception of wavelengths exceeding 920 nm. For the shorter wavelengths between 730 nm and 800 nm, L-cone sensitivity for single-photon absorption is still relatively high compared to wavelengths beyond 800 nm [2]. At the same time, 2P absorption excites S-cones at half of the laser wavelength, which is between 365 nm and 400 nm in this range. That means, that the sensitivity for 2P absorption is below the maximum absorption of the S-cone photopigment at around 420 nm [28], while 1P absorption still works rather effectively. From 850 nm, 2P excitation takes place at ∼ 425 nm well inside the visible range of the spectrum and sensitivity for 1P stimulation is reduced by about 6 magnitudes compared to the peak of L-cones at around 560 nm [29]. Approximated sensitivity curves can be found in Fig. 1.

With mean shifts in complementary wavelength of 15 to 51 nm between measurements, we observe large shifts in perceived color, indicating strong effects of both laser power and repetition frequency. While an increase in power causes a linear increase in 1P excitation, the probability of 2P excitation exhibits quadratic proportions to average laser power (see Eq. (1)). This leads to a shift in 1P/2P ratio benefiting 2P absorption, thus causing a blue shift in perceived color. This nonlinear relation leads to the fact that the mix of 1P and 2P vision does not obey Grassmann’s laws [30], as scalar properties are not given. Changing intensity affects the two mechanisms to a different degree, and color constancy with a 1P metamer does not persist. When changing repetition frequency while keeping average laser power constant, there is no effect in 1P absorption to be expected, whereas there is a reciprocal connection between repetition frequency and 2P excitation. Again, the ratio of 1P/2P absorption shifts, resulting in a change in color perception. While there is little variability in the colorshifts between 730 nm and 800 nm, the area of rivaling 1P and 2P excitation exhibits very high variability. This is in agreement with previous studies observing a high variability in colormatches at wavelengths lower than 950 nm [20]. Possible explanations include imperfect alignment of participants to the system, resulting in a slight defocus/ decentering of the laser beam, the presence of high-order aberrations in the eyes tested, even though the small beam diameter should limit the effects of high-order aberrations, and optical differences in individual correction as well as differences at the photoreceptor level. Prior work also suggested individual differences in macular pigment concentration as a source of high intersubject variability in the perception of blue 2P stimulation. Postreceptoral amplification factors that normally compensate for the attenuation of blue light by macular pigment and differ in strength depending on pigment density might also amplify 2P signals that are unaffected by this process [31,32]. As 2P absorption heavily relies on a high photon density at the photoreceptor level, every aspect negatively impacting the quality of the laser’s PSF on the retina will diminish the efficiency of 2P absorption, although small values of defocus were shown to not significantly impact visibility thresholds [33]. While this will only result in a lower brightness and larger spot size in areas of dominant 1P or 2P vision, the area of mixed 1P/2P perception will also exhibit a changed ratio between both effects, resulting in a shift in color perception. Although it is possible to calculate relative ratios of 2P stimulation between two sets of laser parameters using Eq. (1), large variations between participants and even between measurement repetitions of the same participants prevents the reliable verification of these predictions with the data collected. The use of adaptive optics might reduce these individual deviations, possibly even allowing for a universally valid colormap to be created [31]. Figure 6 illustrates the high intersubject variability as well as a general blue shift for all individuals. Enabling a reliable prediction of colors created from mixed 1P/2P stimuli only from system parameters would be a major step towards developing a 2P retina display [34] as well as detecting abnormal color perception that might be caused by ocular diseases or retinal abnormalities.

A severe limitation of the experiment is the gamut of the OLED screen, which does not allow us to recreate a wide range of colors within the CIE 1931 chromaticity diagram. Colormatches located along the very border of the gamut of the screen suggest that the colors produced by the laser were more saturated than what the screen could reproduce, thus adding difficulty to the colormatching task. Additionally, the stimulation brightness could not be recorded as it was not possible to reproduce the brightness of the laser stimulus on the screen. Participants described all stimuli as very bright and saturated, and we do not expect the differences in brightness between different stimuli to have a strong influence on the color matching results. However, to minimize the impact of those restrictions, complementary wavelength was chosen to quantify shifts in color. While this is not a very common method, this simplified approach focuses on the hue of color matches, without considering saturation or brightness. This way, we do not only focus our attention on the most reliable information in our data, but we also mitigate the influence of the small gamut of the screen that might otherwise interfere with the analysis. By calculating complementary wavelength instead of direct distances between colormatches, we can directly translate our findings to the more uniform CIE 1976 UCS, showing no significant deviations to the calculations in the CIE 1931 color space.

To improve the quality and accuracy of colormatches in the future, the OLED could be replaced with a display providing a larger gamut or even a three channel laser system in the visible range. Picking the right wavelengths for red, green and blue channels would increase the gamut of the matching stimulus significantly and facilitate the matching task for future participants. Interestingly, participants reported that particularly the purple colors could not be reproduced on the screen very well, and that the laser stimulus was much more saturated than the stimulus of the OLED. The reproducibility of saturated purples may be an additional benefit of a 2P retina display that requires further investigation.

## 5. Conclusion

We showed that nonspectral purples can be created using a pulsed laser with only one single NIR wavelength. The range of wavelengths exhibiting a balanced ratio between red 1P stimulation and blue 2P stimulation for this setup can be defined between 850 and 920 nm. Due to the nonlinear properties of 2P vision, both laser power and repetition frequency can be used as parameters to move the color perception towards red or blue, though the ratio between 1P and 2P perception seems to be highly variable between individuals. Applying adaptive optics might decrease these variations significantly while also allowing for larger beam diameters without sacrificing PSF quality at the same time. This could possibly enable NA to be used as another parameter to shift the ratio between 1P and 2P excitation. Additionally, this might allow for a universally valid colormap to be created, enabling reliable prediction of colors created from mixed 1P/2P stimuli.

## Supplemental information

Supplement 1 Detailed visualization of data distribution https://doi.org/10.6084/m9.figshare.30293914

## Data Availability

Data underlying the results presented in this paper are not publicly available at this time but may be obtained from the authors upon reasonable request.

## Supplemental document

See Supplement 1 for supporting content.
