# Supplementary material for: Seeing nonspectral colors with single wavelength stimulation in two-photon vision
Source: Biomed Opt Express. 2025 Oct 30;16(11):4767–76. doi: 10.1364/BOE.575139 (PMC12643018; doi:10.1364/BOE.575139)
Supplement: Supplementary file 1 [file boe-16-11-4767-s001.pdf]

Supplemental Document

Biomedical Optics EXPRESS

Seeing nonspectral colors with single wavelength stimulation in two-photon vision: supplement

LINUS EMMERICH,[1,2,*] PEDRO GIL,[3,4] SILVESTRE MANZANERA,[3,4] JUAN TABERNERO,[3,4] PABLO ARTAL,[4] AND CHRISTINA SCHWARZ[1,5]

[1] *Institute for Ophthalmic Research, University of Tübingen, Tübingen, Germany*
[2] *Graduate Training Center of Neuroscience, University of Tübingen, Tübingen, Germany*
[3] *Electromagnetismo y Electrónica, Universidad de Murcia, Murcia, Spain*
[4] *Laboratorio de Óptica, Universidad de Murcia, Murcia, Spain*
[5] *Center for Optical Technologies, Aalen University, Aalen, Germany*
[*] *linus.emmerich@uni-tuebingen.de*

This supplement published with Optica Publishing Group on 30 October 2025 by The Authors under the terms of the Creative Commons Attribution 4.0 License in the format provided by the authors and unedited. Further distribution of this work must maintain attribution to the author(s) and the published article's title, journal citation, and DOI.

Supplement DOI: https://doi.org/10.6084/m9.figshare.30293914

Parent Article DOI: https://doi.org/10.1364/BOE.575139

Seeing nonspectral colors with single wavelength stimulation in two-photon vision: supplemental document

1. DATA VISUALIZATION

To provide a more detailed insight into the measurements, Figures S1-S7 show the distribution of data points separated for each stimulation wavelength. Different markers indicate different combinations of laser powers and repetition frequencies.

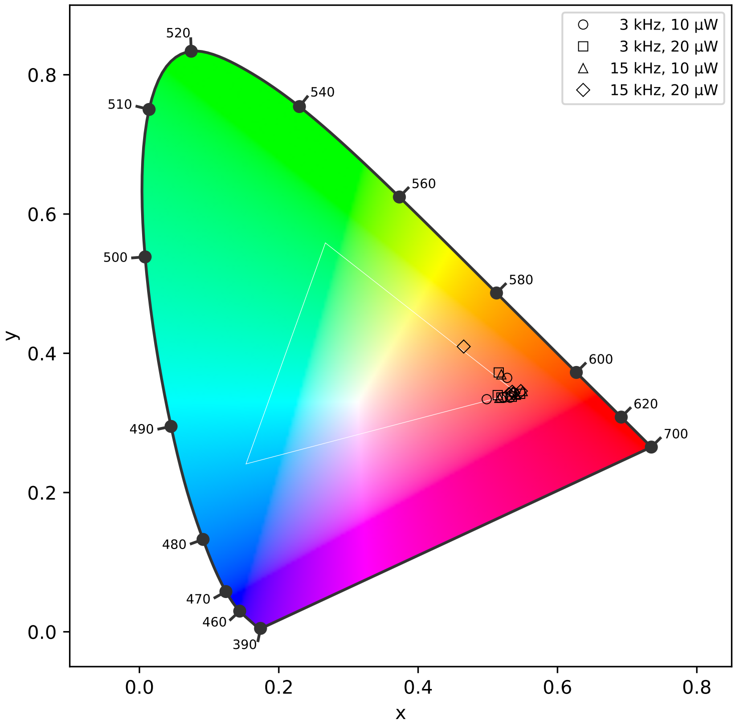

Fig. S1. Distribution of data points for a stimulation wavelength of 730nm.

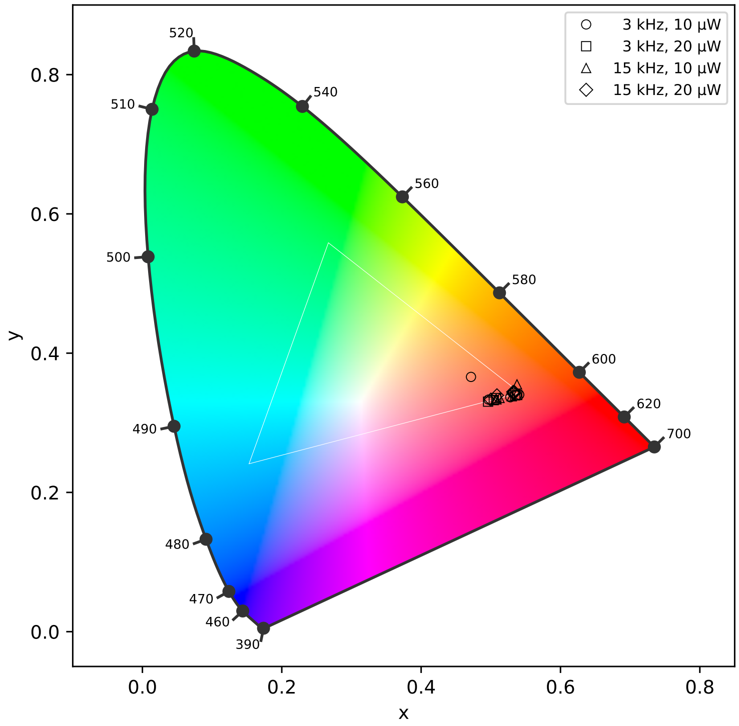

Fig. S2. Distribution of data points for a stimulation wavelength of 760nm.

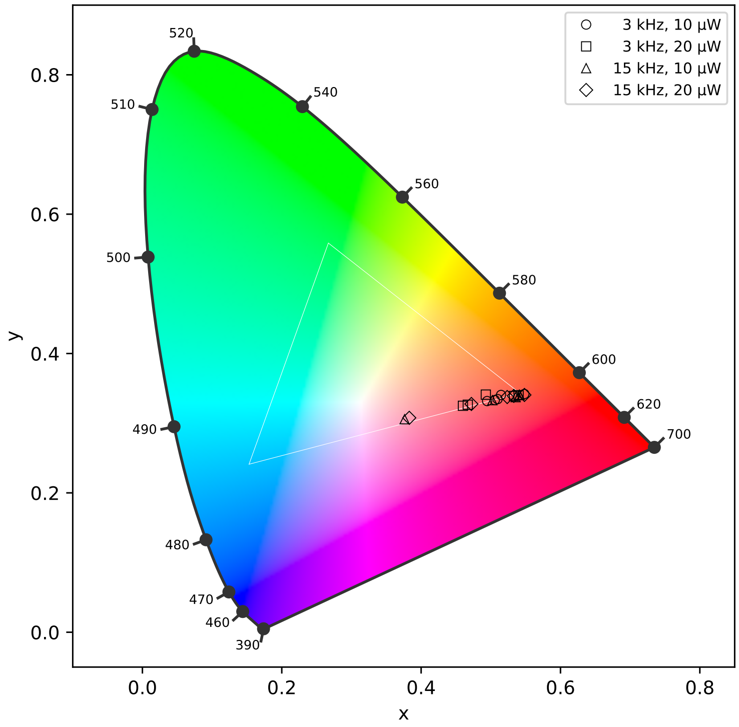

Fig. S3. Distribution of data points for a stimulation wavelength of 800nm.

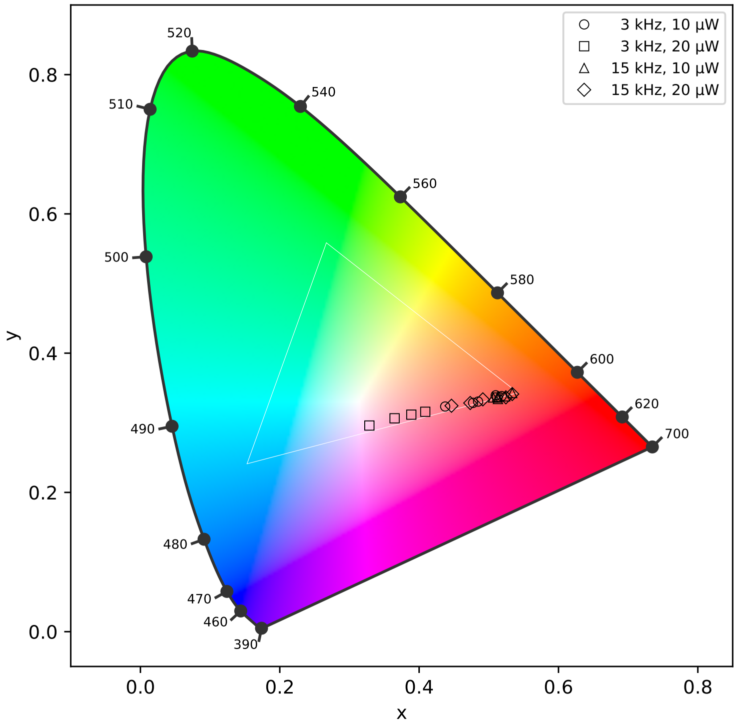

Fig. S4. Distribution of data points for a stimulation wavelength of 850nm.

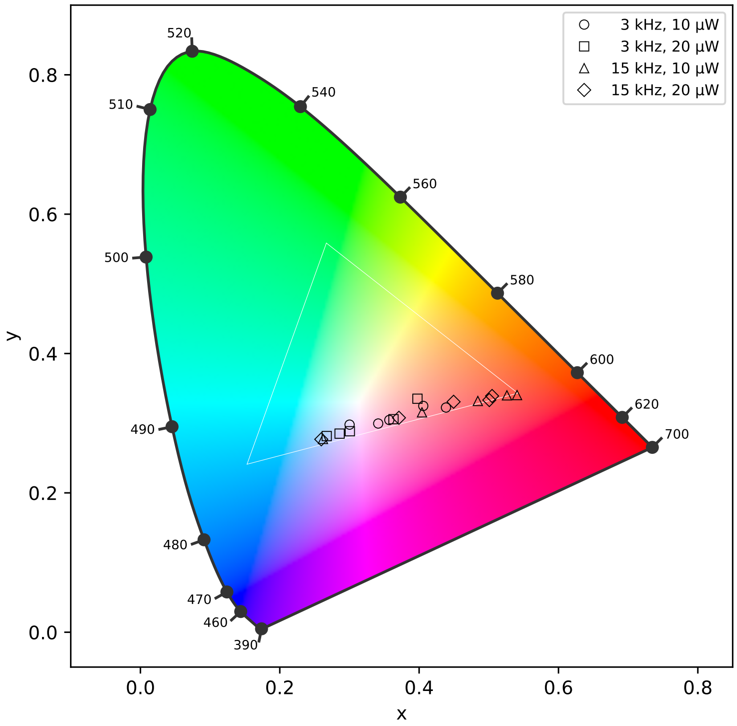

Fig. S5. Distribution of data points for a stimulation wavelength of 880nm.

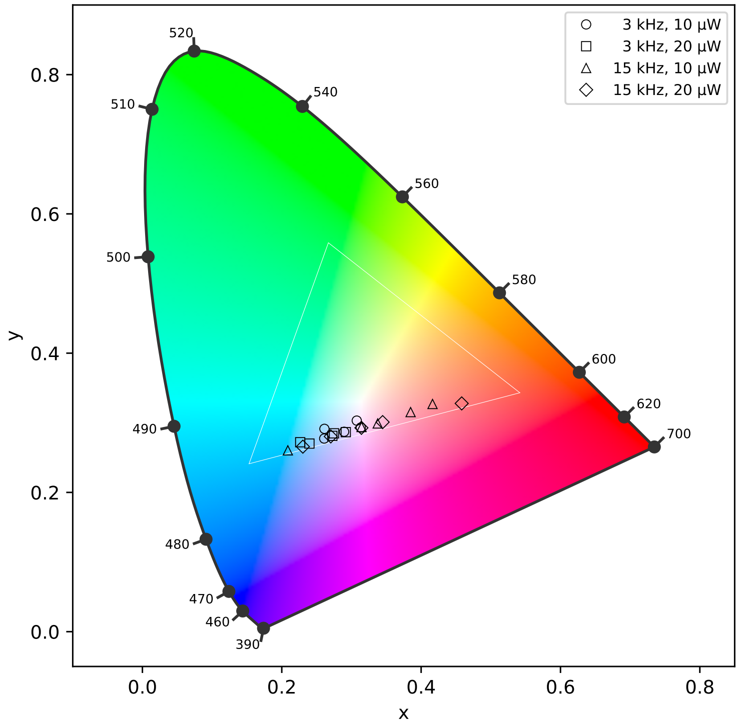

Fig. S6. Distribution of data points for a stimulation wavelength of 900nm.

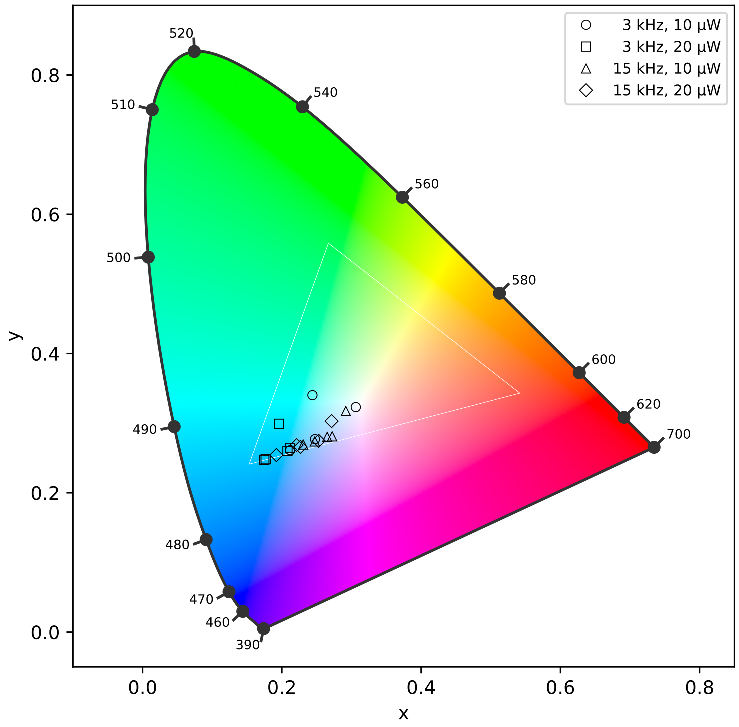

Fig. S7. Distribution of data points for a stimulation wavelength of 920nm.
